# A proposal for monitoring the process of internalization following Galperin's conception

**DOI:** 10.3389/fpsyg.2023.1152541

**Published:** 2023-05-12

**Authors:** Leonardo Daniel Rivera Valdez, Vicente Arturo López Cortés, Marco Antonio García Flores

**Affiliations:** Facultad de Psicología, Benemérita Universidad Autónoma de Puebla, Puebla, Mexico

**Keywords:** private speech, internalization, activity theory, cultural-historical psychology, developmental psychology

## Abstract

Since the era of Piaget and Vygotsky, private speech (PS) has been widely discussed, but in recent years, the avenues for its study have greatly expanded. In this study, we explored the use of a recoding scheme for PS inspired by the studies of Pyotr Galperin. A coding scheme for social speech, PS, and the lack of speech, as the form of action (FA) has been proposed (i.e., external social speech, external audible speech, inaudible speech, and mental FA when no speech was produced). An exploratory study was conducted to elucidate the appropriateness of the coding scheme, both ontogenetically and during tasks. The results showed that both the coding scheme by type of speech and FA were adequate for differentiating ontogenetically between children. However, only the coding schemes of the FA were appropriate for differentiating between children as a function of their performance (i.e., time and scores) in a Tower of London task. Moreover, Galperin's scheme was more suitable when there was redundancy in performance between those with audible and inaudible external speech.

## Introduction: Vygotsky and the internalization process

The process of internalizing speech was deeply studied and theorized by Vygotsky (2012). He proposed that private speech (PS) was an intermediate step between social speech and inner speech, but he also attributed it a role in self-regulating activity. For him regulation first occurred due to the influence of adults in social speech and later transferred to self-regulation because of PS. Finally, the regulation became internalized in inner speech.

This conception of PS was aligned with his general notion that functions appear two times in development: first in the social space and then in the internal or mental space (Vygotsky, 2012). Such conceptions have been deeply influential in studying the process of the genesis of self-regulation in preschool children (see Winsler, 2009).

One of the most influential coding schemes for studying this process of internalization was proposed by Berk (1986). In this coding scheme, one first needs to separate the utterances produced by the child in the condition selected by the experimenter (e.g., play) according to temporal and semantic criteria (Winsler et al., 2005). Then, one divides the speech according to whether it is social or PS. Social speech is coded when there is physical or visual contact, when the context refers to someone or something that was said, or when it is temporarily related to the speech of another individual. Everything else is considered PS. Further, PS is classified as follows: (1) level 1 if PS (PS1) is irrelevant to the task, word play or repetition, emotional expression irrelevant to the task, or commentaries to absent or imaginary characters; (2) level 2 if PS (PS2) is relevant to the task, describes the child's own activity, is self-guided commentary, is a self-answered question, or is an emotional expression relevant to the task; and (3) level 3 of PS (PS3) if PS is externalized inner speech relevant to the task (e.g., verbal murmurs, whispers, and lip and tongue movements).

To elicit this kind of speech, many conditions have been used, such as naturalistic settings, social settings, free play, planning tasks, constructive tasks, memory tasks, and response inhibition tasks (Berk, 1992; Fernyhough and Fradley, 2005; Winsler et al., 2005; Fernyhough and Meins, 2009; Winsler, 2009). The common denominator is that the task is significant and aligned with the level of development such that it is significantly challenging.

Such a scheme has been highly fruitful not only in studying the process of internalization but also in examining its relationship with other cognitive functions or investigating PS in atypically developing populations (please refer to Winsler, 2009). This scheme has been used to study PS across the lifespan (Berk, 1992; Winsler, 2009). The study on PS in the specific language impairment population (Lidstone et al., 2012) has been productive in studying PS in the ADHD and ASD populations (Winsler et al., 2007). For studying microgenetic (Benigno et al., 2011) and transversal relations between PS and executive functioning (Fernyhough and Fradley, 2005; Alarcón-Rubio et al., 2014; Thibodeaux et al., 2019) as well as the ontogenetic relation between PS and theory of mind (Fernyhough and Meins, 2009; Rivera, 2023), Berk's coding scheme has been highly fruitful. However, it is important to note that Berk's coding scheme is not the only coding scheme (please refer to Winsler et al., 2005) available for studying the internalization process of the self-regulation function.

## Galperin's notion of internalization

Galperin was a member of the Kharkov school of pyschology that was headed by Leontiev and where other important psychologists such as Vygotsky, Luria, Bozhovich, and Zaporozhets worked. He integrated the ideas of internalization of Vygotsky (2012) with the developments of object-centered activity developed by Leontiev (1981). He developed a formative method to study the internalization process (Galperin, 2021). Such a procedure was called the stage-by-stage formation of mental actions. Moreover, such a procedure allowed Galperin to study the formation of mental actions in connection with object-centered activity at the material/materialized level (i.e., actions with concrete objects or their representations), thus informing, in a controlled way, of the stages in the formation of actions.

According to Galperin, an action traverses multiple qualitative changes or forms of action (FA): material/materialized, externalized social speech, silent external speech, and mental FAs (Galperin, 1967, 1992, 2021). At the material/materialized level, people interact with the material supports needed to solve a particular problem. These can include real objects, graphic representations, and, most of the time (in the context of the experiments), an orientation card that helps the learner solve the problem. After this level was accomplished, the next stage (externalized social speech) consisted of the interaction between the learner and the teacher, where they resolved the problem in collaboration, but especially because the teacher oriented the learner in the space problem (e.g., asking for the steps, correcting him, etc.). Later, the learner would orient himself/herself in the problem space using his/her own speech, which would gradually become more and more abbreviated. Finally, the motor aspect of speech would disappear entirely. At this level, Galperin (2009) suggested that, at this point, the learner would operate at the level of meaning or “pure thought,” which he referred to as the mental form of action.

In this study, we indicate that his internalization stages could be implemented in the studies of PS. We also propose a new coding scheme for PS using the internalization forms of action: material/materialized, externalized social speech, silent external speech, and mental.

However, we recommend retaining the PS coding based on Vygotsky (e.g., such as Berk's) but reclassifying it for continued use. This coding scheme can still be useful for ontogenetical purposes, discourse analysis, and content analysis of the utterances, among others, and shedding light on the transitional process from external, inaudible speech form to mental form. Galperin's proposed forms of internalization include material/materialized, externalized social speech, silent external speech, and mental forms of action. We suggest incorporating the specificity of the literature on the types of PS into Galperin's scheme. We also propose recoding the PS of children to incorporate Galperin's notions of internalization.

The classification is proposed as follows: (1) material/materialized when the concrete objects or their representations are used to solve a task; (2) external social speech when the majority of the utterances are of the social type; (3) audible external speech when the majority of the utterances are of the PS2 type; (4) inaudible external speech when the majority of the utterances are of the PS3 type; and (5) or mental when there is one or no event of speech at all while resolving tasks. This classification would add even more specificity to Galperin's conception since he did not consider audible external speech as others have (Berk, 1986; Winsler, 2009: Winsler et al., 2005) and would shed some light on the complex process of passing from audible external speech to a mental form of action.

Because of the previous considerations, an exploratory analysis was performed to discern if the proposed re-coding by the FA is an appropriate categorization for studying the process of internalization across the preschool years. Does the classification of the FA distinguish between different preschool children (e.g., first and second grade of preschool)? Is this classification better in some respects to other kinds of classification of private speech? Is the reclassification by FA redundant, or does it present new information compared to other classifications?

## Methodology

### Participants

Participants from a previous study that were recruited from the preschools “Jardín de Niños General Lázaro Cárdenas del Río” and “Jardín de Niños Salvador Díaz Mirón” in the state of Tlaxcala, Mexico were included in the study if they did not have previous antecedents of a neurological condition or a learning problem reported by their teachers. Consent was obtained from their parents, and, the children and their parents were free to withdraw from participation in the study at any time during the study. Of the 91 children, four left the study, and one was discontinued because his teacher said that the kid was receiving language therapy. The sample consisted of a total of 86 participants: 24 of them were from the first grade of preschool (boys = 9 and girls = 15; mean age = 4 years, SD = 0.257; range: 3.50–4.33 years); 30 from the second grade of preschool (boys = 13 and girls = 17; mean age = 5.02 years, SD = 0.311; range: 4.58–5.41 years); and 32 from the third grade of preschool (boys = 14 and girls = 18; mean age = 5.98 years, SD = 0.279; range: 5.58–6.75 years).

### Procedure

Consent was obtained from the guardian of every child. First, the participants were led to a quiet room provided by the school (e.g., the empty playroom) where different toys for make-believe play were provided (in the case of the play condition). The children then either performed the free play condition in groups of four or performed the ToL task individually. Subsequently, the children received theory of mind tasks as part of a parallel study. Those in preschool 1 took the theory mind task on another day, and those of preschool 2 and 3 on the same day. Finally, after completing the tasks, the children received a gift and sweets that were delivered as a group.

### Private speech coding

We adopted the proposal of Fernyhough and Meins (2009) where videotaped sessions were divided into utterances according to Bakthin's unit of analysis. In this study, the limits of an utterance were demarcated temporarily and semantically, temporarily by units of 2 s of difference, and semantically by changes in the theme of the utterance. Once this was accomplished, utterances were divided according to whether they were social or private, according to Winsler et al. (2005). An utterance was considered social if: (1) visual contact occurred between the participant and another person for at least 2 s while the utterance was produced; (2) contact (e.g., physical, sight, etc.) occurred between the participant and another person for at least 2 s while the utterance was produced; (3) content involved the content of the previous utterance of another person or mentioned them explicitly; and (4) temporarily, the utterance followed, in a time-lapse of < 2 s, the previous utterance of another person. All the other utterances that did not fulfill these conditions were considered PS.

Second, PS utterances were coded following Berk (1986)'s classification: (1) level 1 if PS (PS1) is irrelevant to the task, word play or repetition, emotional expression irrelevant to the task, or commentaries to absent or imaginary characters; level 2 if PS (PS2) was relevant to the task, described the child's own activity and were self-guided commentaries, were self-answered questions, or were emotional expressions relevant to the task; Finally, level 3 of PS (PS3) was coded if externalized inner speech was relevant to the task (e.g., verbal murmurs, whispers, and lip and tongue movements). Finally, a degree of internalization measure was computed by summing the amounts of PS2 and PS3 and dividing it by the amount of time (in minutes) when such utterances were coded (i.e., $\frac{Total\;PS2\;+\;Total\;PS3}{Total\;Time\;\left(min\right)}$; Fernyhough and Meins, 2009; Winsler, 2009).

### Form of action

The recoding scheme for the PS was realized, as stated above in the section above. Material/materialized form was omitted since it was impossible to evaluate such a form in free play or Tower of London conditions. Thus, coding form was stated as external social speech, audible external speech, inaudible external speech, or mental form as a function of the predominant PS types (i.e., social, PS2, PS3, or none). Therefore, if a participant had a frequency of five in PS3, but a frequency of seven in PS2, the audible external speech FA was assigned. When a conflict occurred, such as when PS2 and PS3 were equal, a less internalized form of action was adopted (i.e., PS2).

### Free play

Since the group of first-graders was very young, we followed Fernyhough and Meins (2009) suggestions of recording free play sessions in groups of four kids for a maximum of 16 min. Two cameras were positioned in a silent room provided by the schools. Their speech was coded following the abovementioned coding schemas.

### Tower of London

Following Fernyhough and Fradley (2005), we applied the Tower of London (ToL) to the second and third grades of preschool to elicit their PS. The ToL consists of three pegs and three rings of different colors (e.g., blue, red, and green), one copy for the participant and another for the researcher to model the target of the trial. The experimenter told the participant, “That they need to make sure that their toy looks equal to this one (the model),” presenting them with four different levels (i.e., 2, 3, 4, and 5 moves) of the task. Further, participants were told some rules: (1) they should use one hand only; (2) they cannot move more than one piece at a time; and (3) they cannot leave the pieces on the table and then move another piece, they should place the piece first on the pegs, and then they can move another one. Finally, children are told that “Some children like to talk out loud when they resolve this task, if you want you can talk. While you play, you can talk and say what you want” to encourage children to talk, otherwise they may not talk even if that is helpful for them. The session was recorded and coded as specified before.

## Results

**Figure 1:**
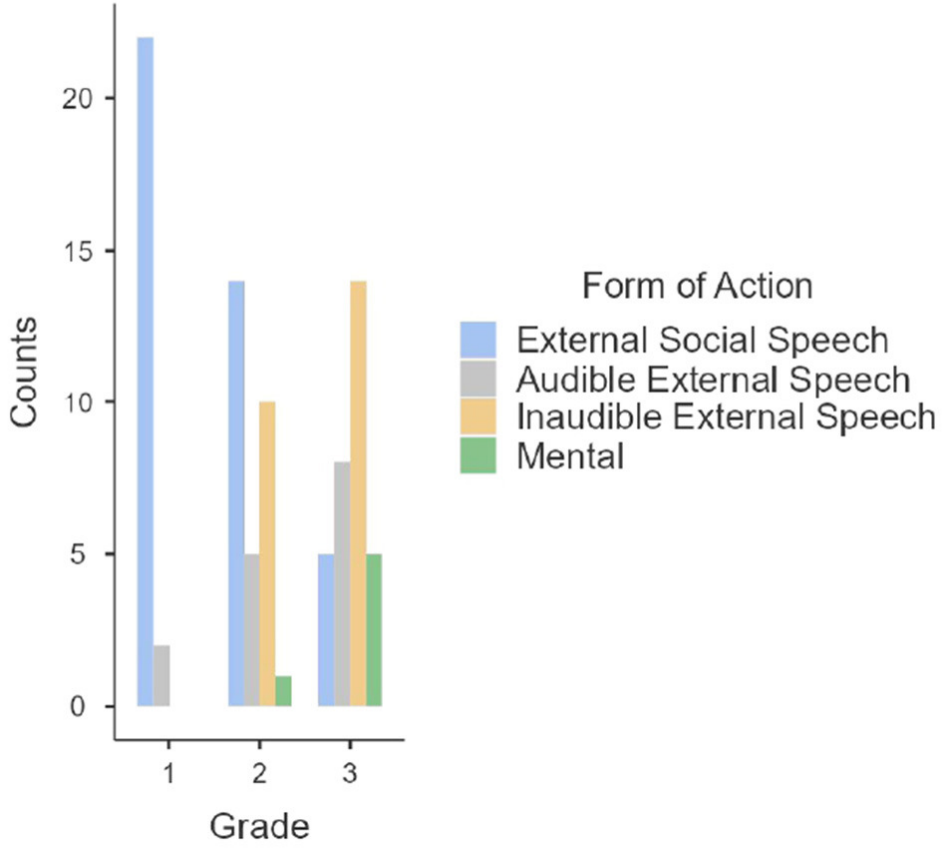
Frequencies of the forms of action.

In Tables 1–3 descriptive statistics of PS, degree of internalization and FA in the preschool grades are presented. Figure 1 presents a graphic of the amount of FA according to the preschool grade. Descriptive statistics showed that children from preschool 1 tend to have a lower degree of internalization than children from preschool 2 and 3. The results showed that preschool 1 only has external social speech FA, while children in preschool 2 have external social speech as the dominant form but also have inaudible external speech, audible external speech, and in last place mental FA; finally, preschool 3 children have as a dominant FA inaudible external speech, audible external speech, mental, and lastly they have external social speech. Therefore, in was not until children reached preschool 3 that inaudible external speech as a dominant form.

**Table 1:** Descriptive statistics of private speech.

	**Grade**	**Social speech**	**PS1**	**PS2**	**PS3**
Mean	Preschool 1	18.9	0.833	3.42	0.0833
	Preschool 2	6.37	0.00	1.70	2.57
	Preschool 3	1.47	0.0625	2.16	2.06
Standard deviation	Preschool 1	12.7	1.86	2.76	0.408
	Preschool 2	8.69	0.00	2.77	2.54
	Preschool 3	2.98	0.354	2.92	2.12

**Table 2:** Descriptive statistics of degree of internalization (DI).

	**Grade**	**DI**
N	Preschool 1	24
	Preschool 2	30
	Preschool 3	32
Missing	Preschool 1	0
	Preschool 2	0
	Preschool 3	0
Mean	Preschool 1	0.262
	Preschool 2	2.19
	Preschool 3	3.09
Standard deviation	Preschool 1	0.207
	Preschool 2	2.07
	Preschool 3	2.07

**Table 3:** Frequencies of FA.

**FA**	**Grade**	**Frequencies**	**% of total**	**Cumulative %**
External social speech	Preschool 1	22	25.6%	25.6%
	Preschool 2	14	16.3%	41.9%
	Preschool 3	5	5.8%	47.7%
Audible external speech	Preschool 1	2	2.3%	50.0%
	Preschool 2	5	5.8%	55.8%
	Preschool 3	8	9.3%	65.1%
Inaudible external speech	Preschool 1	0	0.0%	65.1%
	Preschool 2	10	11.6%	76.7%
	Preschool 3	14	16.3%	93.0%
Mental	Preschool 1	0	0.0%	93.0%
	Preschool 2	1	1.2%	94.2%
	Preschool 3	5	5.8%	100.0%

### Differences in degree of internalization across preschool grades

ANOVA analyses were performed following Wilcox (2017), who recommended the use of trimmed means for incrementing the power of the analyses (for some computational and implementation details, see Mair and Wilcox, 2020; Love and Mair, 2022). The analyses revealed that there were significant differences between preschool groups (*F* = 25.1, *p* < 0.001). *Post-hoc* analyses were conducted (see Table 4), and it was found that the first grade of preschool had a lower degree of internalization than the second grade ($\hat{\psi }$ = −1.52, *p* = 0.002); that the first grade had a lower degree of internalization than the third grade ($\hat{\psi }=$ −2.85, *p* < 0.001); and that second grade had a lower degree of internalization than the third grade ($\hat{\psi }=$ −1.32, *p* = 0.035).

**Table 4:** *Post-hoc* tests—degree of internalization as a function of grade.

		**psi-hat**	***p***	**95% Confidence interval**	
				**Lower**	**Upper**
1	2	−1.52	0.002	−2.51	−0.540
1	3	−2.85	< 0.001	−4.08	−1.608
2	3	−1.32	0.035	−2.83	0.186

1, preschool 1; 2, preschool 2; 3, preschool 3.

### Differences in performance as a function of speech type and FA

**Figure 2:**
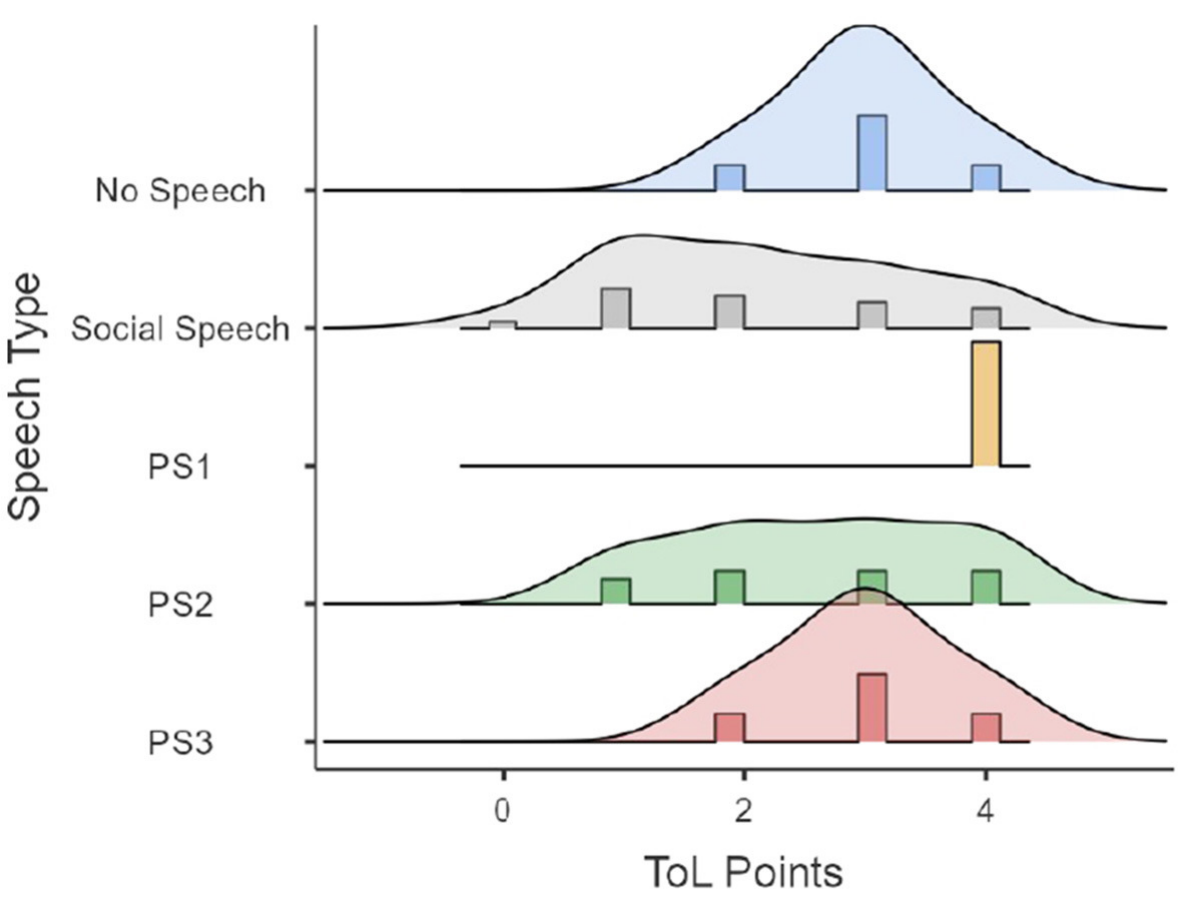
ToL points as a function of speech type.

**Figure 3:**
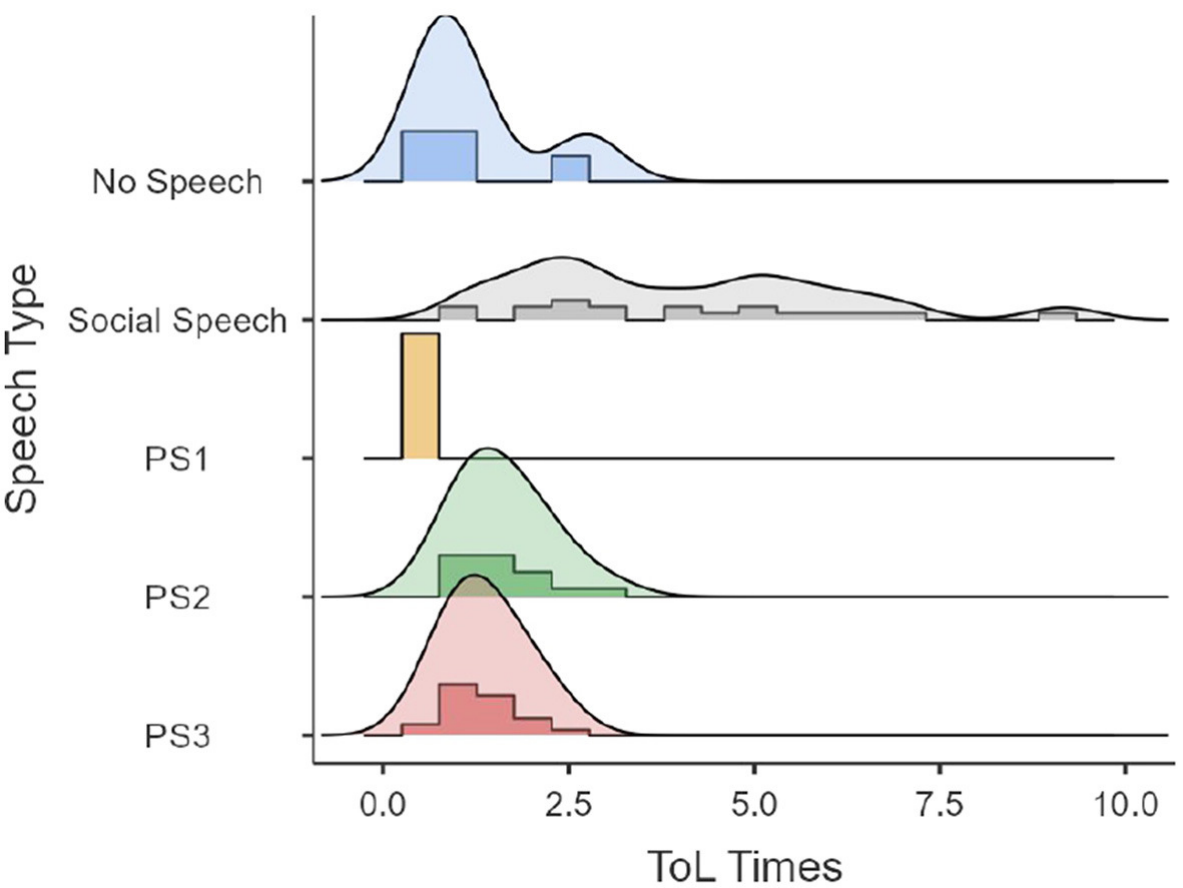
ToL times as a function of speech type.

First, performances of time and ToL points as a function of speech type (i.e., the dominant type for each participant) are presented in Figures 2, 3. Figures 2, 3 show that a lack of speech events is not classifiable with the private speech coding scheme. Figure 2 shows that ToL points as a function of speech type are widely distributed as a function of the speech type and are uninformative. Figure 3 shows that ToL times seem to be widely distributed when the speech type is social but reduced when the speech is more internalized (i.e., PS1, PS2, and PS3).

**Figure 4:**
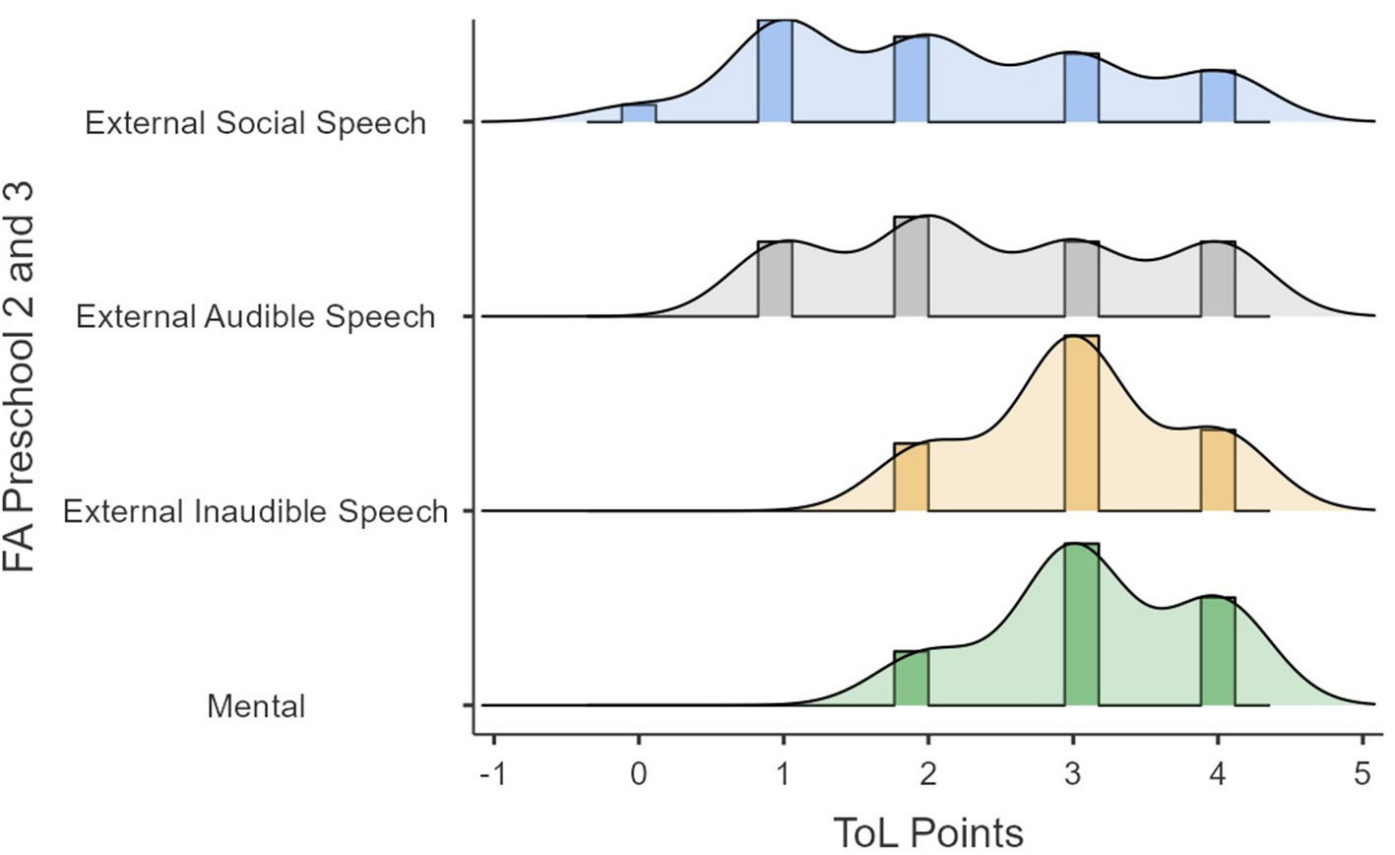
ToL points as a function of FA.

**Figure 5:**
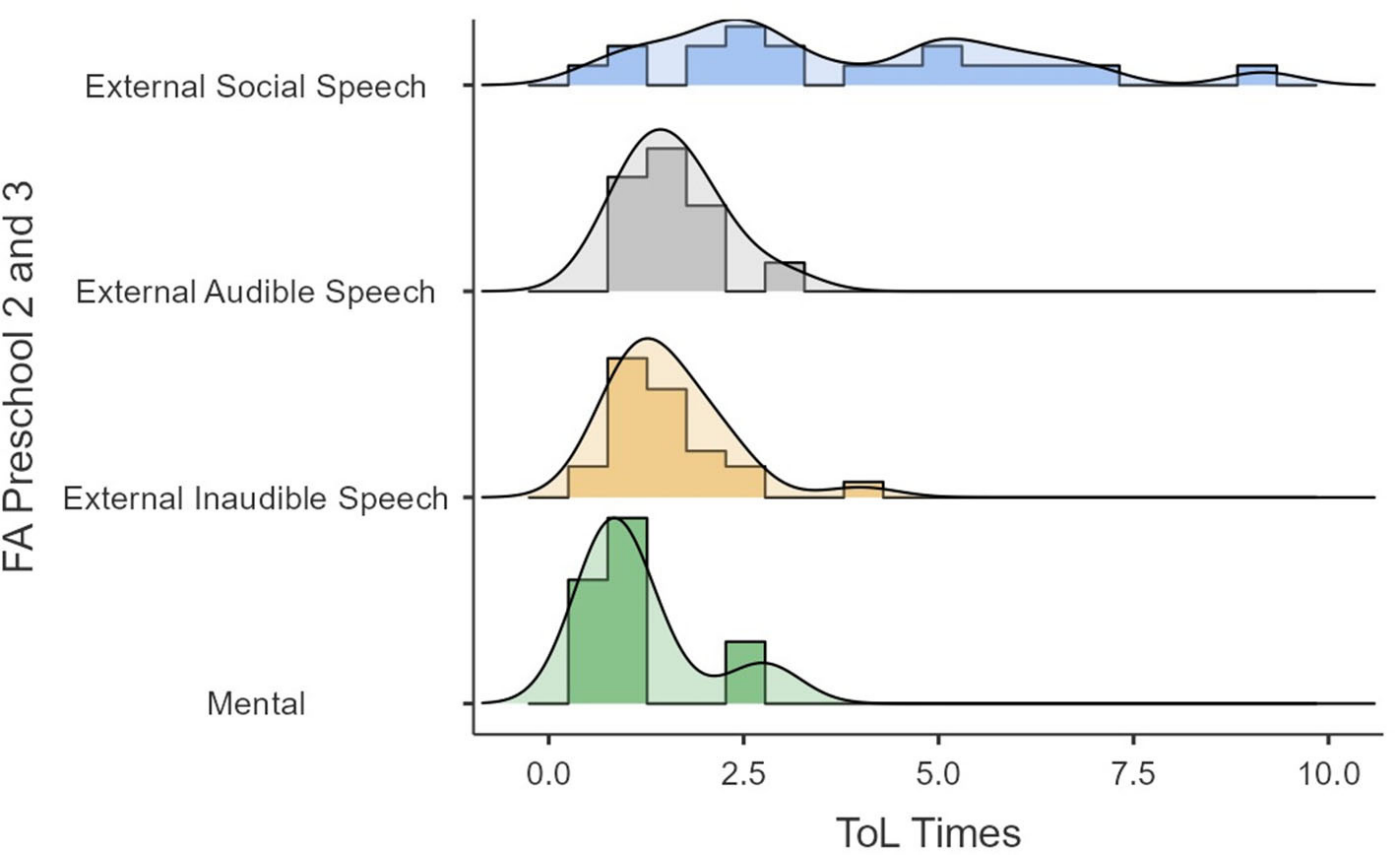
ToL times as a function of FA.

Second, Figures 4, 5 show the differences in times and ToL points as a function of the FA. Figure 4 shows that, as the process of internalization progresses, the dispersion of the ToL scores becomes smaller, and scores tend to be on the higher end. While Figure 5 shows that, as the internalization progresses, the dispersion of times of execution in ToL becomes smaller, especially when FA passes from external social speech to external audible speech.

An analysis of ANOVA for the type of speech with trimmed means was not possible; thus, classical non-parametric tests were performed (i.e., Kruskal–Wallis test). No significant difference was found for ToL points as a function of speech type (χ^2^ = 8.90, df = 4, *p* = 0.064), while a significant difference was found for time (χ^2^ = 29.3, df = 4, *p* < 0.001). Pairwise comparisons (see Table 5) showed that participants with social speech took more time resolving the ToL than those with PS2 (W = −5.28, *p* = 0.002) and PS3 (W = −6.55, *p* < 0.001) types of speech but not more time than those with PS1 type (W = −2.33, *p* = 0.467). Those with PS1 type did not differ from those with PS2 (W = 2.30, *p* = 0.479) or PS3 (W = 2.35, *p* = 0.459) types. Moreover, those with PS2 type did not differ from those with PS3 type (W = −1.69, *p* = 0.756). Finally, those with a lack of speech showed faster executions than those with social speech (W = 3.92, *p* = 0.044), but no difference from those with PS1 (W = −2.07, *p* = 0.586), PS2 (W = 2.22, *p* = 0.515), or PS3 (W = 1.59, 0.793) types.

**Table 5:** Pairwise comparisons—ToL times as a function of speech type.

		**W**	***p***
No speech	Social speech	3.92	0.044
No speech	PS1	−2.07	0.586
No speech	PS2	2.22	0.515
No speech	PS3	1.59	0.793
Social speech	PS1	−2.33	0.467
Social speech	PS2	−5.28	0.002
Social speech	PS3	−6.55	< 0.001
PS1	PS2	2.30	0.479
PS1	PS3	2.35	0.459
PS2	PS3	−1.69	0.756

The ANOVA analyses indicated a significant difference in the score of the ToL (*F* = 3.34, *p* = 0.053). The *post-hoc* analyses (see Table 6) showed that children with an external social speech FA showed lower scores in ToL than those with an inaudible external speech FA ($\hat{\psi }\;=$ −1.063, *p* = 0.050). However, they did not differ from those with an audible external speech FA ($\hat{\psi \;}=$ −0.444, *p* = 0.653) or a mental FA ($\hat{\psi \;}=$ −1.250, *p* = 0.112). Further, those with an audible external speech FA did not differ from those with an inaudible external speech FA ($\hat{\psi \;}=$ −0.618, p=0.653) or a mental FA ($\hat{\psi \;}=$ −0.806, *p* = 0.653). Finally, the group that had an inaudible external speech FA did not differ significantly from those with a mental FA ($\hat{\psi }\;=$ −0.188, *p* = 0.653).

**Table 6:** *Post-hoc* tests—ToL scores of preschool 2 and 3 as a function of FA.

		**psi-hat**	***p***	**95% Confidence interval**	
				**Lower**	**Upper**
1	2	−0.444	0.653	−2.09	1.20580
1	3	−1.063	0.050	−2.12	−0.00933
1	4	−1.250	0.112	−2.74	0.23738
2	3	−0.618	0.653	−2.20	0.96016
2	4	−0.806	0.653	−2.60	0.98538
3	4	−0.188	0.653	−1.66	1.28227

1, External social speech; 2, external audible speech; 3, external inaudible speech; 4, Mental.

The ANOVA analysis of time in function of the FA was also significant (*F* = 7.82, *p* = 0.002). The *post-hoc* analyses (see Table 7) showed that those with an external social speech FA consumed a significant amount of time to resolve the ToL task than those with an audible external speech FA ($\hat{\psi }\;=$ 2.107, *p* = 0.013), those with an inaudible external speech FA ($\hat{\psi }\;=$ 2.225, *p* = 0.010), or those with a mental FA ($\hat{\psi }\;=$ 2.723, *p* = 0.003). However, those with an audible external speech FA did not differ significantly from those with an inaudible external speech FA ($\hat{\psi }\;=$ 0.118, *p* = 0.588) or a mental FA ($\hat{\psi }\;=$ 0.616, *p* = 0.068). Finally, those with an inaudible speech FA did not differ from those with a mental FA ($\hat{\psi }$ = 0.498, *p* = 0.083).

**Table 7:** *Post-hoc* tests—ToL times of preschool 2 and 3 as a function of FA.

		**psi-hat**	***p***	**95% Confidence interval**	
				**Lower**	**Upper**
1	2	2.107	0.013	0.320	3.895
1	3	2.225	0.010	0.448	4.003
1	4	2.723	0.003	0.936	4.511
2	3	0.118	0.588	−0.507	0.743
2	4	0.616	0.068	−0.117	1.350
3	4	0.498	0.083	−0.189	1.185

1, External social speech; 2, external audible speech; 3, external inaudible speech; 4, Mental.

### Differences in performance as a function of FA according to Galperin's categories of internalization

**Figure 6:**
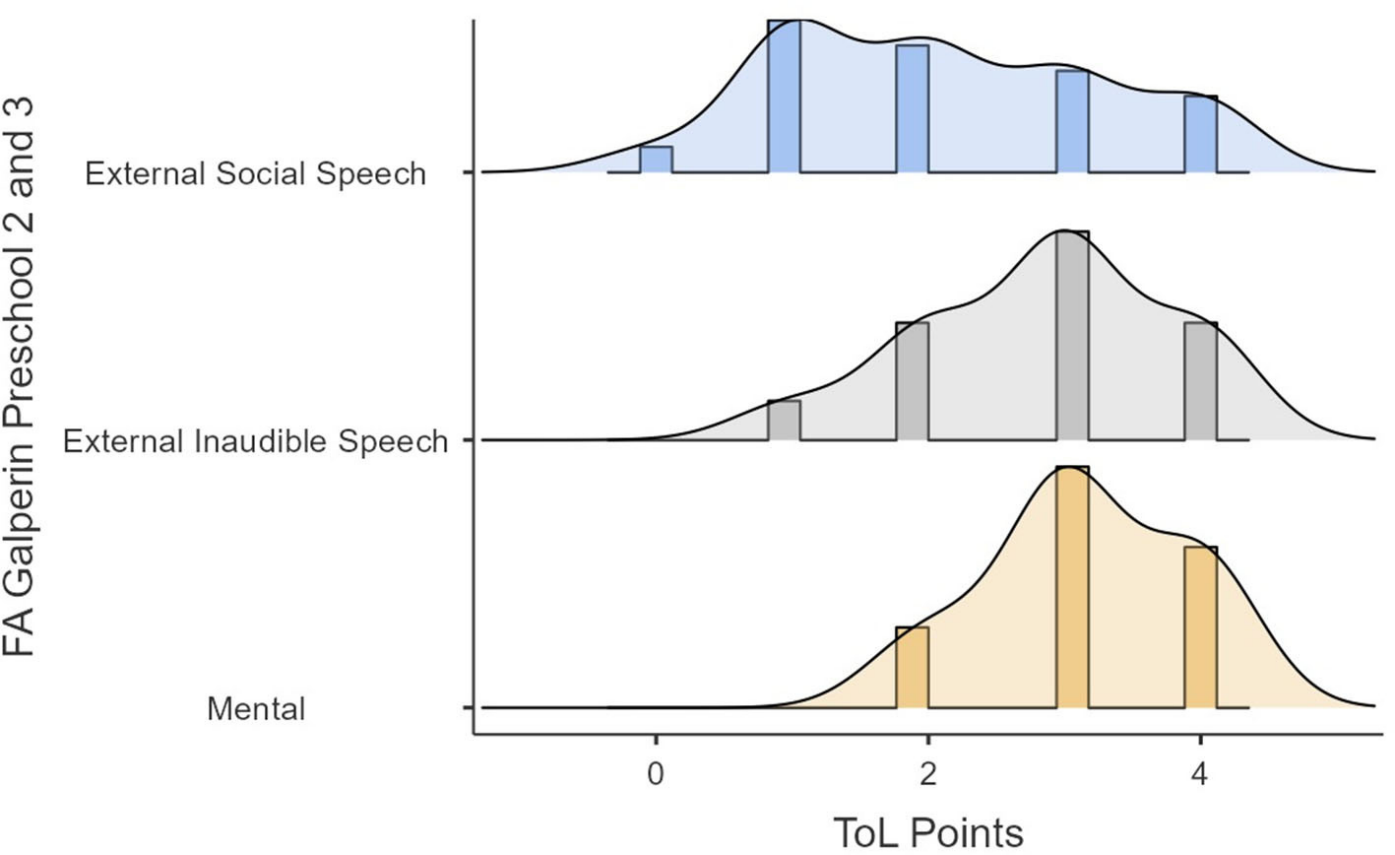
ToL points as a function of FA.

**Figure 7:**
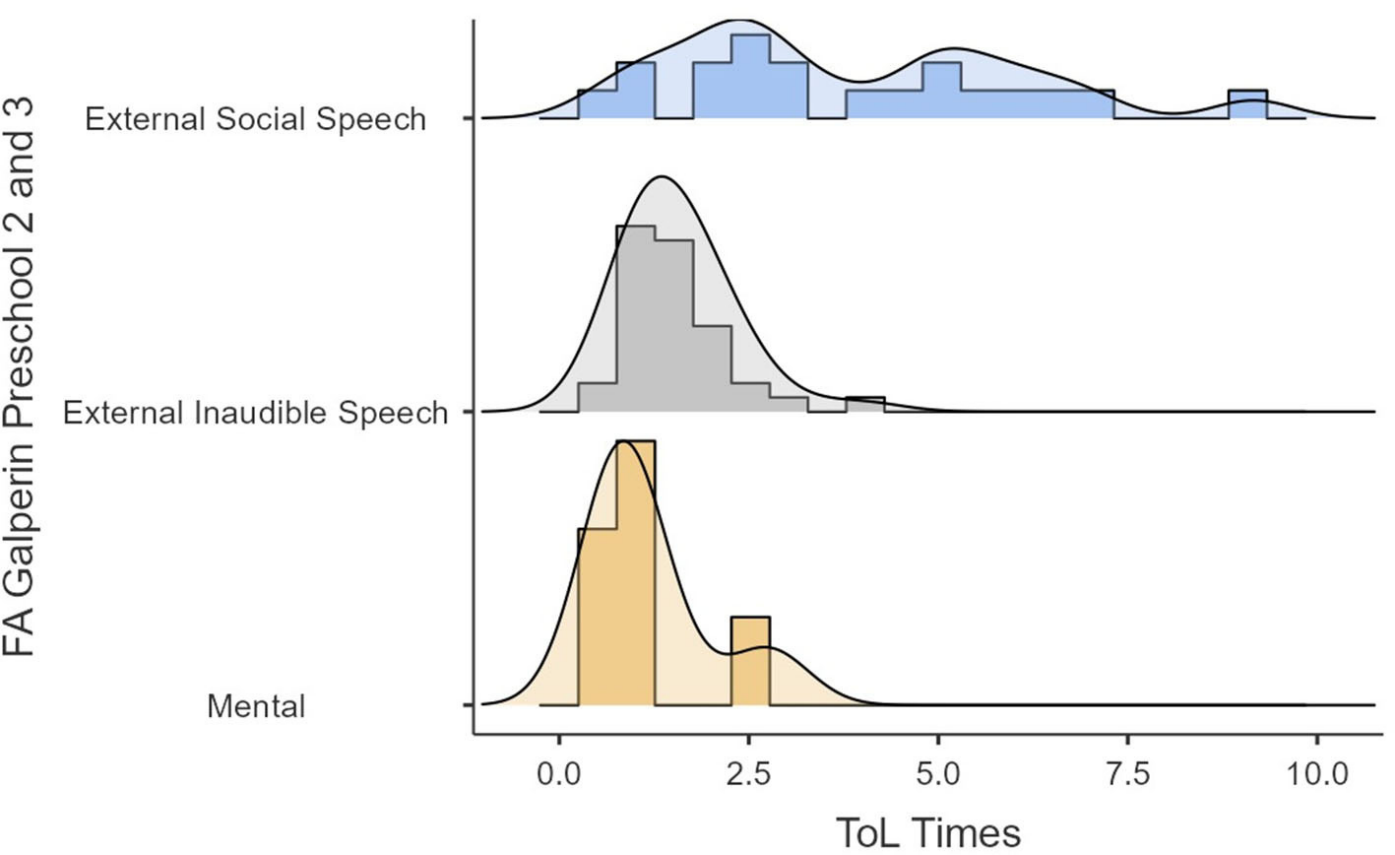
ToL times as a function of FA.

The previous analyses showed redundancy between audible and inaudible external speech that the mental FA showed a tendency toward significance, and that the type of speech tended to be a variable that did not capture the differences in performance well. Because of these, the same analyses were reproduced but with Galperin's categories of internalization. This indicates that we did not consider the differences between audible external speech and inaudible external speech to oneself. Figures 6, 7 show the performances in time and points in the ToL as a function of FA (i.e., external social speech, inaudible external speech, and mental). Figures 6, 7 show how the variance of the times and scores in ToL tend to be lower as the internalization process progresses.

Significant differences were found in ToL scores (*F* = 4.15, *p* = 0.045). In concrete terms, *post-hoc* analyses showed (please refer to Table 8) that those with an external social speech FA had lower scores than those with an external inaudible speech ($\hat{\psi \;}=$ −0.870, *p* = 0.049) or a mental FA ($\hat{\psi \;}=$ −1.250, *p* = 0.049). However, there was no difference in performance between those with an external inaudible and a mental FA ($\hat{\psi \;}=$ −0.380, *p* = 0.370).

**Table 8:** *Post-hoc* tests of ToL scores with respect to Galperin's coding scheme in preschool 2 and 3.

		**psi-hat**	***p***	**95% Confidence interval**	
				**Lower**	**Upper**
External social speech	External inaudible speech	−0.870	0.049	−1.80	0.0588
External social speech	Mental	−1.250	0.049	−2.56	0.0622
External inaudible speech	Mental	−0.380	0.370	−1.66	0.8976

Finally, the ANOVA analysis showed a significant difference in times for the ToL task as a function of the FA (*F* = 11.7, *p* = 0.002). The *post-hoc* analyses (see Table 9) showed those with an external social speech were slower at solving the ToL task than those with an external inaudible speech ($\hat{\psi }$ = 2.194, *p* = 0.005) and a mental FA ($\hat{\psi \;}\;=$2.723, *p* = 0.001). Moreover, the participants with externally inaudible speech were slower than those with a mental FA ($\hat{\psi }\;=$ 0.530, *p* = 0.029).

**Table 9:** *Post-hoc* tests of time in the ToL with respect to Galperin's coding scheme in preschool 2 and 3.

		**psi-hat**	***p***	**95% Confidence interval**	
				**Lower**	**Upper**
External social speech	External inaudible speech	2.194	0.005	0.6180	3.77
External social speech	Mental	2.723	0.001	1.1312	4.32
External inaudible speech	Mental	0.530	0.029	−0.0622	1.12

## Discussion

The results showed that the degree of internalization was increasing significantly across preschool grades, which is consistent with previous results in the literature (Fernyhough and Meins, 2009; Winsler, 2009). Further, the findings revealed that speech type was not a satisfactory category for studying the changes in performance across ages. Some types of speech were not effective in determining the performance of the task. Categorization by speech type was not able to show differences in scores, but it did show that those with predominantly PS2 and PS3 types were faster than those with social speech. Nonetheless, the degree of internalization was important for differentiating the children at different ages. Therefore, it is an important marker of the ontogenetic development of children.

The categories of speech presented us with the problem of what to do with the cases where kids tend not to talk. This was especially true for older children, but when we collected data, it was clear that even some younger kids seemed to have little to no speech. Because of that, it was critical to consider other aspects, such as the relationship between performance and lack of speech. The latter point is important since many kids may not want to talk even if prompted to do so, as suggested by Fernyhough and Fradley (2005). This could be for many reasons, such as unfamiliarity with the researcher, embarrassment, cultural reasons (e.g., that you should not talk to strangers), and anxiety about tests.

Further, FA coding schemes solved the problem of the unclassifiable lack of speech and also showed to be effective in identifying differences between participants even without the consideration of the material/materialized in this study; older children tended to have inaudible external speech and audible external speech FA while younger children had mainly external social speech. FA classification showed that children with FA that signaled a more internalized action had better performances in time and scores during ToL tasks. The last point was especially clear when we considered the coding scheme of Galperin himself rather than our own, in which we attempted to consider the more subtle forms of PS in the literature (see Winsler et al., 2005; Winsler, 2009). Galperin's account ignored the differences between PS relevant to the task and those not and seemed to ignore the subtleties between audible and non-audible external speech. Ignoring such subtleties seemed correct when we were concerned with the performance in the ToL task (i.e., time and score). Our coding scheme was not effective in determining changes in scores and only revealed that those with external social speech had lower scores than those with inaudible external speech. Galperin's approach, on the contrary, revealed that those with external social speech had lower scores than those with inaudible external speech and mental FA. Then, when we considered the time of execution, our approach determined that those with an external social speech FA took longer to solve the ToL task than those with an external audible, inaudible, or mental FA. However, Galperin's account found that both (i.e., external inaudible speech and mental FA) consumed less time to resolve the ToL task and that those with a mental FA resolved faster than those with an external inaudible form of action.

The last point is consistent with Galperin's approach. He proposed that, in the last stages of internalization, one of the specific changes that occur is automatization of action. This is consistent with the fact that the main difference between mental and inaudible external speech is in time but not in scores of ToL (Galperin, 2021).

Therefore, our results suggest that classifications based on the internalization of actions are a more suitable scheme for classifying the internalization process than speech-type classifications, at least when performance is an issue. This does not imply that such schemes are not useful. On the contrary, we believe that such classification schemes may be useful when the content of the speech is relevant or when the subtleties of PS are of interest. We also believe that the degree of internalization markers is an important quantitative measure for making ontogenetic claims about the internalization process. Nonetheless, we believe that the categories of the FA may be more useful when we want to consider changes in performance since it seems that further subdivisions of speech are redundant for performance (i.e., scores and time). These categorizations would be of great use in neuropsychological neurorehabilitation, where the monitoring of the process is crucial, and the focus is on performance during microgenetic processes.

One concern that the reader may have, as some reviewers pointed out, is that Galperin's approach is normally associated with such formative experiments. This is study is not one of those studies. We agree with them, Galperin is specially associated with that, and that aspect should not be ignored but embraced since it is a very rich description of how to form an action. Nonetheless, we believe that Galperin's intentions were broader. His purpose was to describe the process of internalization broadly, and he found in such a formative method a fruitful way to approach the problem in a controlled manner (Arievitch and Van der Veer, 1995). Because of that, the study of how to apply his categories to ontogeny is valid, and it is in need of more research. However, as we mentioned earlier, we believe that such a process is going to be useful, and is going to be fruitful in such contexts where formation of actions is needed (e.g., neuropsychological rehabilitation; see Engeness and Lund, 2020). For such purposes, we believe that our coding scheme is going to be very useful since it provides a systematic way of studying such formative processes.

The limitations of the study are clear. Since it was only an exploratory study, no clear answers on the topic can be made presently. However, it will be a task of the future to explore the utility of these classifications in other practical cases, especially in the neuropsychological rehabilitative and pedagogical processes.

## Conclusion

This exploratory study successfully showed that PS recoding is a valuable addition to the study of internalization. Coding by the FA proved to be effective in distinguishing between children of different ages and levels of internalization. Further, this coding scheme was useful for differentiating between children according to their time and score performances, which is contrary to the type of speech classification. The latter was not sensitive and was inadequate for those children who performed better but did not speak. As mentioned, this re-coding scheme seems promising for studies that need to monitor the changes in performance as a function of time, teaching, and a rehabilitation process. Future studies will clarify the utility of these coding schemes.

## Data availability statement

The raw data supporting the conclusions of this article will be made available by the authors, without undue reservation.

## Ethics statement

The studies involving human participants were reviewed and approved by Facultad de Psicología BUAP. Written infor med consent to participate in this study was provided by the participants' legal guardian/next of kin.

## Author contributions

LR conceived the initial idea, recollected, transcribed and analyzed the data, and wrote the manuscript. LR and VL discussed and reviewed the theory, methodology, and the final manuscript. VL and MG gathered the resources for publishing and obtained the location for gathering data. All authors contributed to the article and approved the submitted version.
