# Corrigendum: A proposal for monitoring the process of internalization following Galperin's conception

**DOI:** 10.3389/fpsyg.2023.1221706

**Published:** 2023-06-30

**Authors:** Leonardo Daniel Rivera Valdez, Vicente Arturo López Cortés, Marco Antonio García Flores

**Affiliations:** Facultad de Psicología, Benemérita Universidad Autónoma de Puebla, Puebla, Mexico

**Keywords:** private speech, internalization, activity theory, cultural-historical psychology, developmental psychology

In the published article, the inclusion of the separate references, “Vygotsky (2012a)” and “Vygotsky (2012b)”, was incorrect. Instead, all in-text citations should be written as “Vygotsky (2012)” and linked to the following reference details: Vygotsky, L. S. (2012). *Obras Escogidas II: Pensamiento y lenguaje*. Machado Nuevo Aprendizaje. The reference “Vygotsky, L. S. (2012b). The science of psychology. J. Russian East Eur. Psychol. 50, 85–106. doi: 10.2753/RPO1061-0405500404” was not used in the text and should be deleted from the reference list.

In the published article, there were also errors in the text. The word “speech” was incorrectly added to the mental form of action. A correction has been made to the *Abstract*. The corrected *Abstract* is shown below.

Since the era of Piaget and Vygotsky, private speech (PS) has been widely discussed, but in recent years, the avenues for its study have greatly expanded. In this study, we explored the use of a recoding scheme for PS inspired by the studies of Pyotr Galperin. A coding scheme for social speech, PS, and the lack of speech, as the form of action (FA) has been proposed (i.e., external social speech, external audible speech, inaudible speech, and mental FA when no speech was produced). An exploratory study was conducted to elucidate the appropriateness of the coding scheme, both ontogenetically and during tasks. The results showed that both the coding scheme by type of speech and FA were adequate for differentiating ontogenetically between children. However, only the coding schemes of the FA were appropriate for differentiating between children as a function of their performance (i.e., time and scores) in a Tower of London task. Moreover, Galperin's scheme was more suitable when there was redundancy in performance between those with audible and inaudible external speech.

Additionally, there were errors in the **Introduction**. Firstly, the intention was to say that Vygotsky attributed a self-regulation role to private speech. A correction has been made to the section **Introduction: Vygotsky and the internalization process**, Paragraph 1. The corrected paragraph is shown below:

The process of internalizing speech was deeply studied and theorized by Vygotsky (2012). He proposed that private speech (PS) was an intermediate step between social speech and inner speech, but he also attributed it a role in self-regulating activity. For him regulation first occurred due to the influence of adults in social speech and later transferred to self-regulation because of PS. Finally, the regulation became internalized in inner speech.

Secondly, the text of **Introduction**, Paragraph 3, is incorrect. It says that the lack of commentaries should be classified as private speech of type 1, while it should have said that speech that was directed to an absent character should be coded as private speech of type 1. A correction has been made to the section **Introduction: Vygotsky and the internalization process**, Paragraph 3. The corrected paragraph is shown below.

One of the most influential coding schemes for studying this process of internalization was proposed by Berk (1986). In this coding scheme, one first needs to separate the utterances produced by the child in the condition selected by the experimenter (e.g., play) according to temporal and semantic criteria (Winsler et al. 2005). Then, one divides the speech according to whether it is social or PS. Social speech is coded when there is physical or visual contact, when the context refers to someone or something that was said, or when it is temporarily related to the speech of another individual. Everything else is considered PS. Further, PS is classified as follows: (1) level 1 if PS (PS1) is irrelevant to the task, word play or repetition, emotional expression irrelevant to the task, or commentaries to absent or imaginary characters; (2) level 2 if PS (PS2) is relevant to the task, describes the child's own activity, is self-guided commentary, is a self-answered question, or is an emotional expression relevant to the task; and (3) level 3 of PS (PS3) if PS is externalized inner speech relevant to the task (e.g., verbal murmurs, whispers, and lip and tongue movements).

Additionally, the text should have expressed that the objective was to explore if the re-coding by the form of the action was effective at discriminating between the different groups of preschool. A correction has been made to the section **Galperin's notion of internalization**, Paragraph 6. The corrected paragraph is shown below.

Because of the previous considerations, an exploratory analysis was performed to discern if the proposed re-coding by the FA is an appropriate categorization for studying the process of internalization across the preschool years. Does the classification of the FA distinguish between different preschool children (e.g., first and second grade of preschool)? Is this classification better in some respects to other kinds of classification of private speech? Is the reclassification by FA redundant, or does it present new information compared to other classifications?

As in the error above, the text should have said that the speech that was directed to absent characters should be considered private speech of type 1. Additionally, the word “separated” was used incorrectly here, since the calculation of the degree of internalization is $\frac{Total\;PS2\;+\;Total\;PS3}{Total\;Time\;\left(min\right)}$ so the correct action should have been to divide. Corrections have been made to section **Methodology**, *Private speech coding*, Paragraph 2. The corrected paragraph is shown below.

Second, PS utterances were coded following Berk (1986)'s classification: (1) level 1 if PS (PS1) is irrelevant to the task, word play or repetition, emotional expression irrelevant to the task, or commentaries to absent or imaginary characters; level 2 if PS (PS2) was relevant to the task, described the child's own activity and were self-guided commentaries, were self-answered questions, or were emotional expressions relevant to the task; Finally, level 3 of PS (PS3) was coded if externalized inner speech was relevant to the task (e.g., verbal murmurs, whispers, and lip and tongue movements). Finally, a degree of internalization measure was computed by summing the amounts of PS2 and PS3 and dividing it by the amount of time (in minutes) when such utterances were coded (i.e., $\frac{Total\;PS2\;+\;Total\;PS3}{Total\;Time\;\left(min\right)}$; Fernyhough and Meins, 2009; Winsler, 2009).

In addition to this, the correct intention was to state that the room was provided by the school, not that the cameras and the room were provided by us. A correction has been made to the section **Methodology**, *Free play*. The corrected section is shown below.

Since the group of first-graders was very young, we followed Fernyhough and Meins (2009) suggestions of recording free play sessions in groups of four kids for a maximum of 16 min. Two cameras were positioned in a silent room provided by the schools. Their speech was coded following the abovementioned coding schemas.

The text also should have said that there were two copies of the Tower of London. One for the children, and another one for the researchers to show the target model of the trial. Additionally, it should have expressed that they cannot move and leave the piece on the table. A correction has been made to the section **Methodology**, *Tower of London*. The corrected section is shown below.

Following Fernyhough and Fradley (2005), we applied the Tower of London (ToL) to the second and third grades of preschool to elicit their PS. The ToL consists of three pegs and three rings of different colors (e.g., blue, red, and green), one copy for the participant and another for the researcher to model the target of the trial. The experimenter told the participant, “That they need to make sure that their toy looks equal to this one (the model),” presenting them with four different levels (i.e., 2, 3, 4, and 5 moves) of the task. Further, participants were told some rules: (1) they should use one hand only; (2) they cannot move more than one piece at a time; and (3) they cannot leave the pieces on the table and then move another piece, they should place the piece first on the pegs, and then they can move another one. Finally, children are told that “Some children like to talk out loud when they resolve this task, if you want you can talk. While you play, you can talk and say what you want” to encourage children to talk, otherwise they may not talk even if that is helpful for them. The session was recorded and coded as specified before.

Additionally, there was an error in the section **Results**. It should have been expressed that the second grade had a lower degree of internalization than the third grade.

A correction has been made to the section **Results**, *Differences in degree of internalization across preschool grades*. The corrected section is shown below.

ANOVA analyses were performed following Wilcox (2017), who recommended the use of trimmed means for incrementing the power of the analyses (for some computational and implementation details, see Mair and Wilcox, 2020; Love and Mair, 2022). The analyses revealed that there were significant differences between preschool groups (*F* = 25.1, *p* < 0.001). *Post-hoc* analyses were conducted (see Table 4), and it was found that the first grade of preschool had a lower degree of internalization than the second grade ($\hat{\psi }$ = −1.52, *p* = 0.002); that the first grade had a lower degree of internalization than the third grade ($\hat{\psi }=$ −2.85, *p* < 0.001); and that second grade had a lower degree of internalization than the third grade ($\hat{\psi }=$ −1.32, *p* = 0.035).

The text is also incorrect with respect to the differences between the Tower of London times. As the statistic shows, there was no difference between those with PS2 and PS3 types. A correction has been made to the section **Results**, *Differences in performance as a function of speech type and FA*, Paragraph 3. The corrected paragraph is shown below.

An analysis of ANOVA for the type of speech with trimmed means was not possible; thus, classical non-parametric tests were performed (i.e., Kruskal–Wallis test). No significant difference was found for ToL points as a function of speech type (χ^2^ = 8.90, df = 4, *p* = 0.064), while a significant difference was found for time (χ^2^ = 29.3, df = 4, *p* < 0.001). Pairwise comparisons (see Table 5) showed that participants with social speech took more time resolving the ToL than those with PS2 (W = −5.28, *p* = 0.002) and PS3 (W = −6.55, *p* < 0.001) types of speech but not more time than those with PS1 type (W = −2.33, *p* = 0.467). Those with PS1 type did not differ from those with PS2 (W = 2.30, *p* = 0.479) or PS3 (W = 2.35, *p* = 0.459) types. Moreover, those with PS2 type did not differ from those with PS3 type (W = −1.69, *p* = 0.756). Finally, those with a lack of speech showed faster executions than those with social speech (W = 3.92, *p* = 0.044), but no difference from those with PS1 (W = −2.07, *p* = 0.586), PS2 (W = 2.22, *p* = 0.515), or PS3 (W = 1.59, 0.793) types.

Lastly, in the original article, there was a mistake in the Figures 4–7 as published. The y-axis text should have said “FA Preschool 2 and 3” for Figures 4, 5, and “FA Galperin Preschool 2 and 3”, for Figures 6, 7. In addition, due to this amendment the following text in the caption of Figures 4, 5 is not required and has been removed: “1, external social speech; 2, external audible speech; 3, external inaudible speech; 4, mental”. The corrected Figures 4–7 appear below.

**Figure 4:**
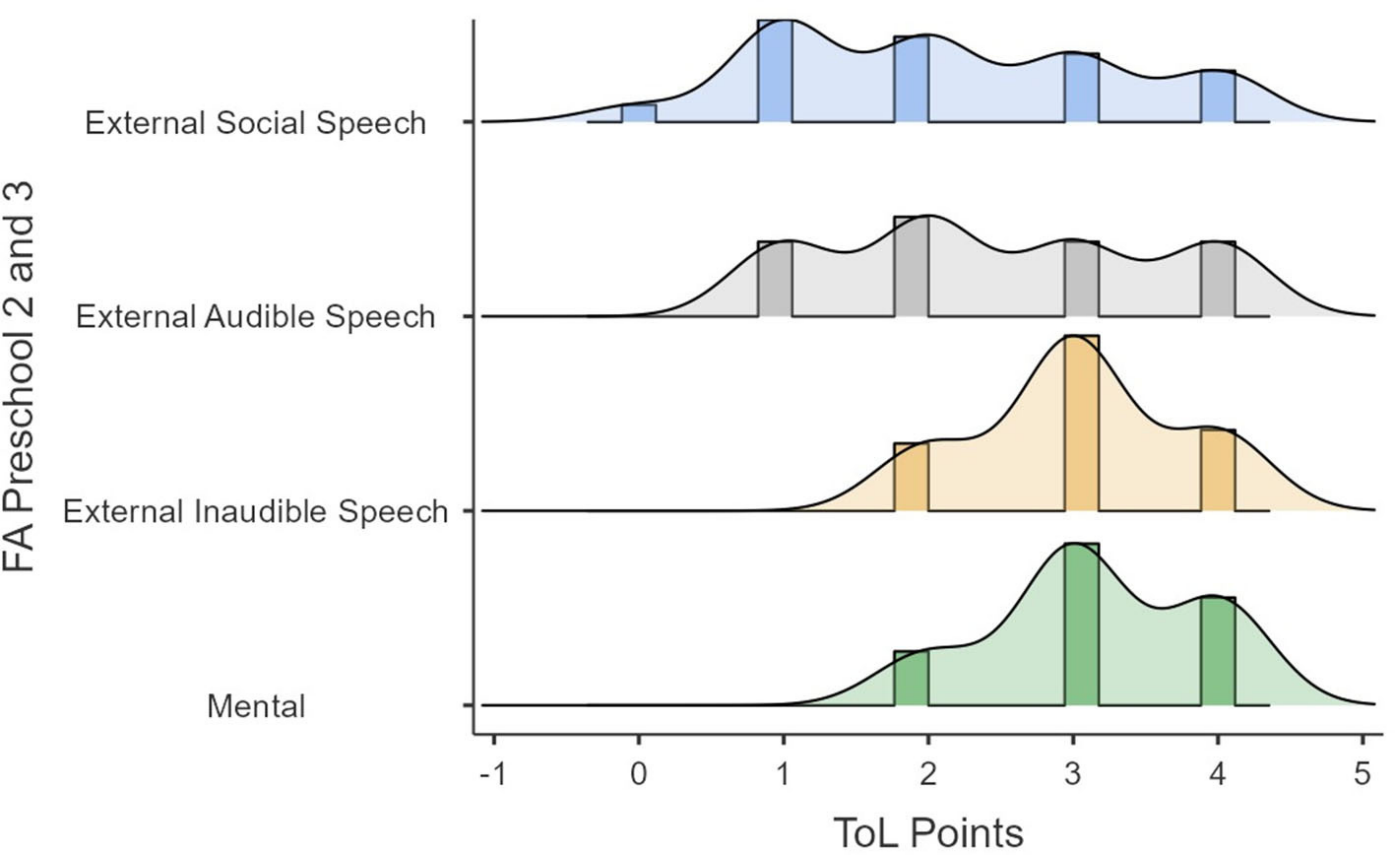
ToL points as a function of FA.

**Figure 5:**
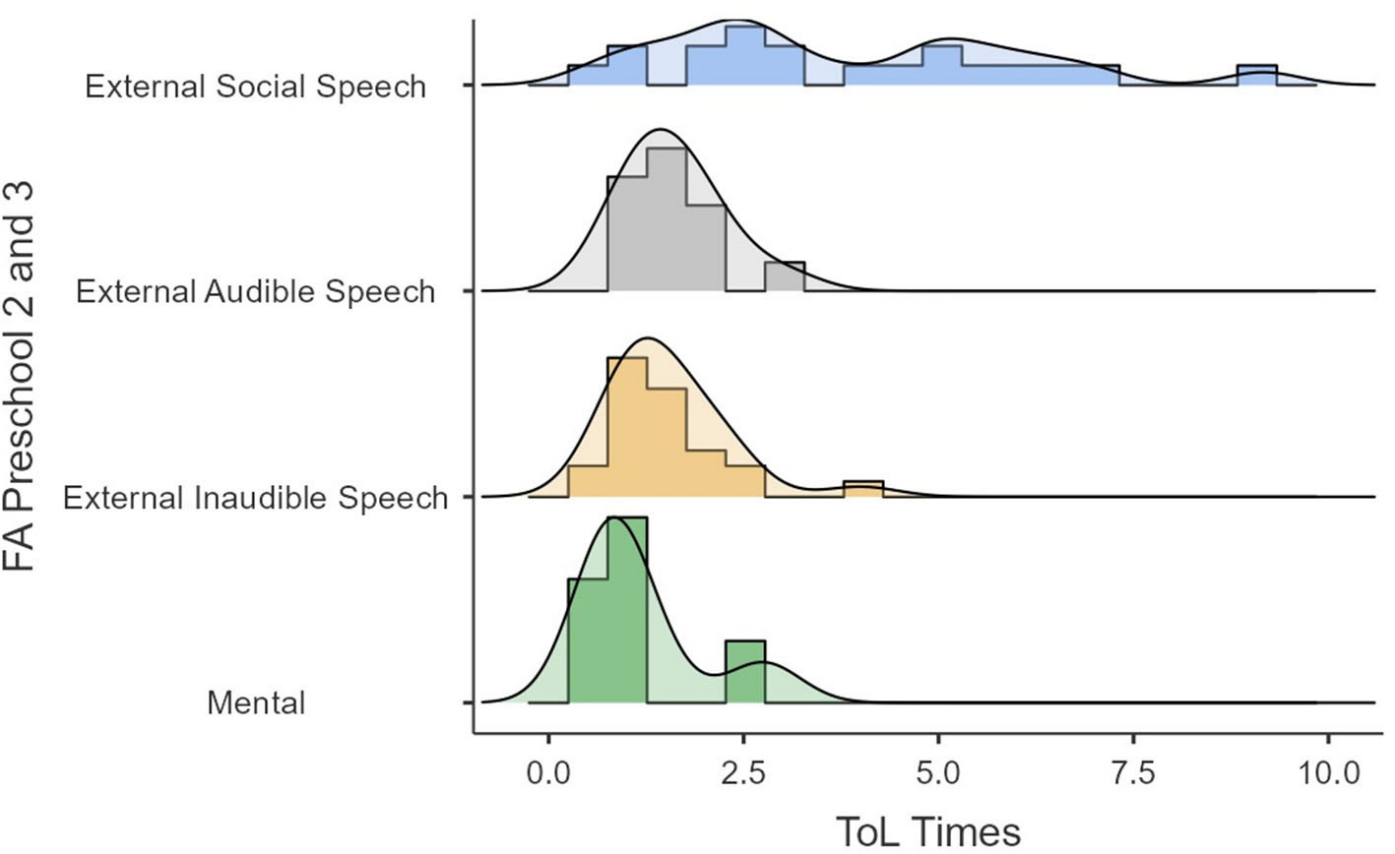
ToL times as a function of FA.

**Figure 6:**
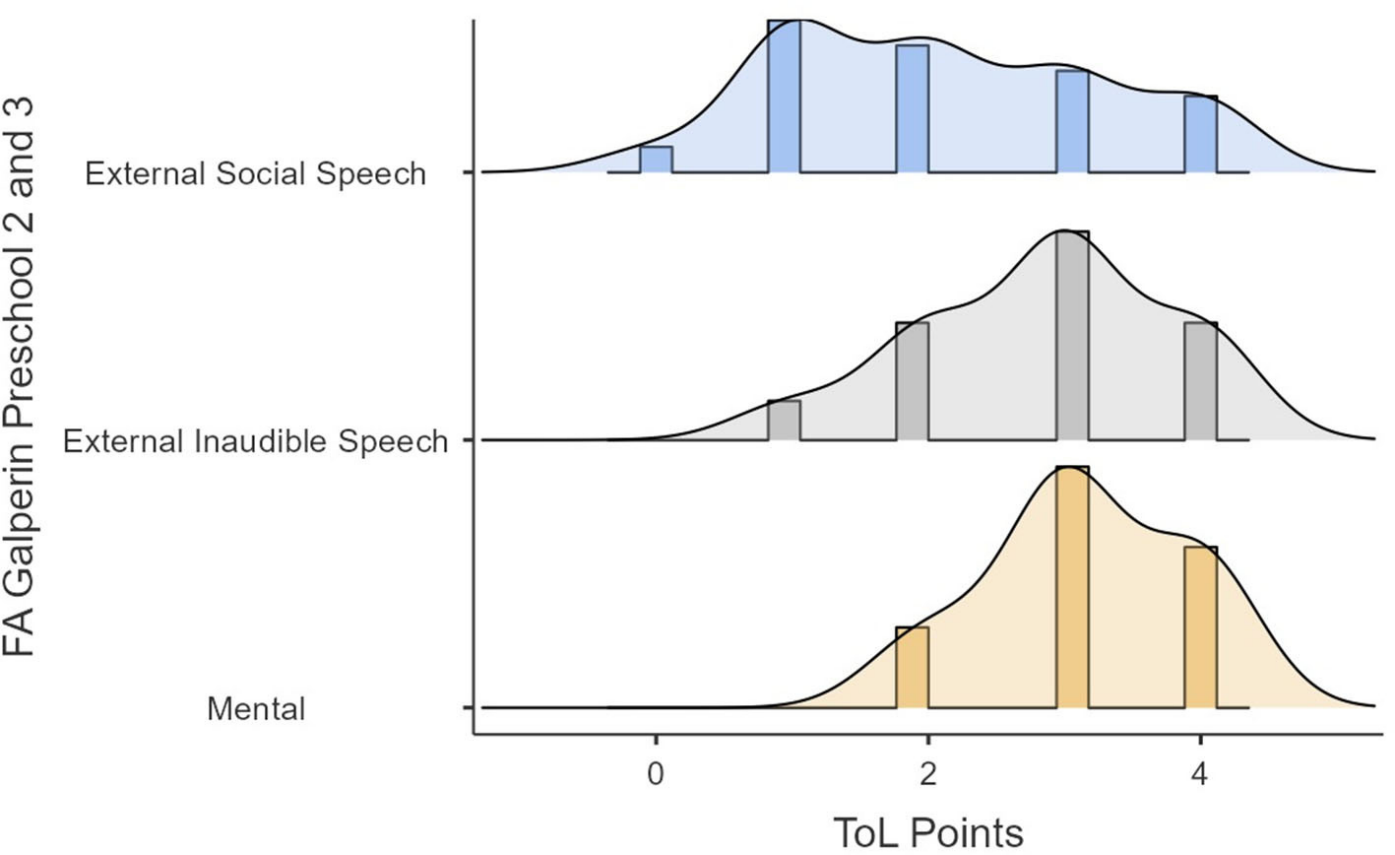
ToL points as a function of FA (Galperin).

**Figure 7:**
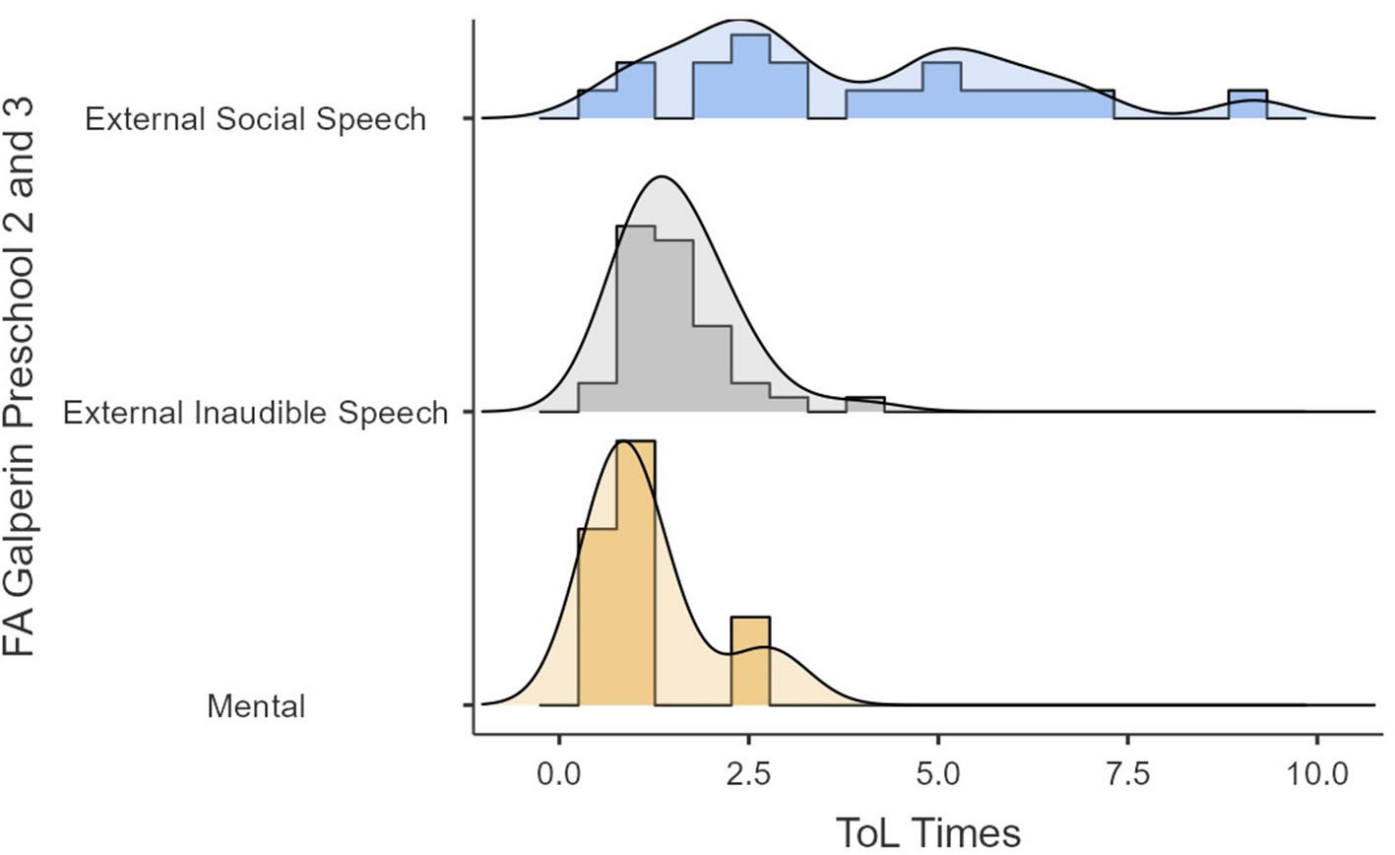
ToL times as a function of FA (Galperin).

The authors apologize for these errors and state that they do not change the scientific conclusions of the article in any way. The original article has been updated.
